# Association between nutritional status and biochemical markers among hematopoietic stem cell transplant candidates: a cross-sectional study

**DOI:** 10.1186/s40795-023-00809-7

**Published:** 2023-12-14

**Authors:** Hoda Zahedi, Sayeh Parkhideh, Omid Sadeghi, Mahshid Mehdizadeh, Elham Roshandel, Makan Cheraghpour, Abbas Hajifathali, Mahdi Shadnoush

**Affiliations:** 1 Hematopoietic Stem Cell Research Center, Shahid Beheshti University of Medical Sciences, Tehran, Iran; 2 Food Security Research Center, Department of Community Nutrition, School of Nutrition and Food Science, Isfahan University of Medical Sciences, Isfahan, Iran; 3 Basic and Molecular Epidemiology of Gastrointestinal Disorders Research Center, Research Institute for Gastroenterology and Liver Diseases, Shahid Beheshti University of Medical Sciences, Tehran, Iran; 4 Department of Clinical Nutrition and Dietetics, National Nutrition and Food Technology Research Institute, Faculty of Nutrition Sciences and Food Technology, Shahid Beheshti University of Medical Sciences, Tehran, Iran

**Keywords:** Nutritional status, Blood biomarkers, Inflammation, BMI, HSCT

## Abstract

**Aim:**

Candidates of Hematopoietic Stem Cell Transplantation (HSCT) may be at nutritional risk due to decreased oral intake, high nutritional requirements and nutrient malabsorption. The aim of this study was to evaluate the association between nutritional status and blood biomarkers in candidates of HSCT.

**Methods:**

A total of 278 patients aged 18–65 years old were recruited and their baseline demographic and clinical characteristics were recorded. All subjects underwent nutritional status analysis using Nutritional Risk Screening (NRS-2002). Blood biomarkers including C-reactive protein (CRP), Erythrocyte Sedimentation Rate (ESR), hemoglobin, albumin and total protein as well as CRP-albumin ratio (CAR) and Body Mass Index (BMI) were measured and compared between two groups based on Nutritional Risk Screening (NRS-2002) within 24 h of admission in Bone Marrow Transplant ward.

**Results:**

The results showed that undernourished patients (NRS ≥ 3) had significantly higher inflammatory markers including ESR, CRP and CAR as well as lower BMI and serum albumin and hemoglobin concentrations (P < 0.05); however, no significant association was observed in terms of total protein even after adjusting for confounders (P > 0.05).

**Conclusions:**

This study revealed that BMI combined with biochemical markers are the appropriate parameters for assessment of nutritional status in HSCT candidates. Furthermore, the nutritional status was verified to be significantly associated with systematic inflammation.

## Introduction

Hematopoietic stem cell transplantation (HSCT) is a well-established medical procedure for treatment of several malignant and benign hematological diseases [1]. Approximately, 50 thousand people undergo HSCT every year worldwide [2]. About 40% of patients experience serious post-transplant complications specifically rapid impairment of nutritional status associated with poor outcome such as higher complication rates during treatment, relapse and overall, lower survival in transplanted patients [3, 4]. Deterioration of nutritional status is an independent risk factor influencing on patients’ quality of life.^(5)^ Therefore, patients undergoing HSCT may be at nutritional risk due to decreased oral intake, high nutritional requirements and nutrient malabsorption [5].

On the other hand, the dramatic induction of local and systemic inflammation resulted from procedural requirements of HSCT plays a vital role in nutritional status [6, 7]. Therefore, initial assessment of nutritional status is important and can be conceived in relation to probable nutritional impacts of conditioning and other treatment-related toxicity [8]. For this purpose, several screening tools have been developed and validated [9]. Of these, the Nutritional Risk Screening (NRS-2002), recommended by the European Society of Clinical Nutrition and Metabolism (ESPEN), has been identified as a well-established tool for the inpatient population associated with higher risk for adverse outcomes. In addition, several studies have shown that inflammation and poor nutritional status evaluated by such parameters as NRS-2002, serum C-reactive protein (CRP) and CRP-albumin ratio (CAR) are in association with poor outcome after HSCT in adults [10, 11]. Instead of analyzing each element separately, CRP and albumin levels will be analyzed together by CAR [12]. The CAR, reflecting both nutritional status and inflammation, has received considerable attention as a novel prognostic parameter in several types of cancers [13–16]. Multiple cancer studies have shown that pre-transplant CAR, a composite index of statistical inflammation and nutritional condition, is an independent predictive predictor. However, its application in patients with hematological malignancies is unknown [17].

The correlation between the nutritional status and systemic inflammation has been investigated in chronic undernourished patients; however, its effect has not yet been evaluated in patients undergoing HSCT. Herein, we hypothesized that an elevated nutritional risk, as assessed by the NRS, is associated with an increased inflammation and lower albumin and total protein levels. In order to test this hypothesis, we performed a cross sectional study aimed to investigate the potential association between the nutritional status and inflammation as well as albumin levels in HSCT Recipients.

## Methods

### Study design and participants

Patients admitted to bone marrow transplant ward of Taleghani Hospital (Tehran, Iran) in order to receive HSCT were identified. Type of hematological malignancies including Multiple Myeloma (MM), Hodgkin Lymphoma (HL), Non-Hodgkin Lymphoma (NHL), Acute Myeloid Leukemia (AML) and Acute Lymphocytic Leukemia (ALL) were confirmed based on pathological findings. The aims and procedures of the study were explained for eligible patients. Of these, 278 adult patients aged 18–65 years old who had signed written consent form were recruited. The patients were included of 99 patients with MM, 55 patients with HL, 28 patients with NHL, 61 patients with AML and 35 patients with ALL. The present study was performed during August, 2020-November 2021 in accordance with the ethical standards of declaration of Helsinki and its later amendments.

### Measurements

All the measurements were implemented within the first 24 h of bone marrow transplant ward admission. Demographic characteristics of the patients including age, sex, diagnosis, type of stem cell transplantation as well as laboratory tests were recorded. Anthropometric data including weight and height were also measured. Weight was measured with minimal clothing and without shoes with 0.1 kg accuracy. Standing height of the patients was measured without shoes with 0.1 cm accuracy (Balas Company, Iran). Body Mass Index (BMI) was calculated by dividing the weight (kg) by height squared (m^2^).

NRS-2002 Questionnaire, used for nutritional assessment, was scored in each of three components including nutritional status of the patient (based on weight loss, Body Mass Index (BMI) and general condition of food intake) and severity of disease and age. Each component is scored from 0 to 3 points and patients aged 70 or older, will receive an extra point [18].

Serum levels of Albumin and total protein were measured using photometric method with a commercial kit (Pars Azmoon Co., Tehran, Iran. Serum concentrations of inflammatory biomarkers including CRP and Erythrocyte Sedimentation Rate (ESR) were measured by immunoturbidimetric assay with a commercial kit (Pars Azmoon Co., Tehran, Iran) and Westergren method, respectively. CAR was calculated by dividing serum CRP concentration by albumin concentration [19]. Serum concentration of hemoglobin also were measured using spectrophotometric method. All assays were performed based on the manufacturer’s procedure.

### Blood sampling

In order to perform laboratory analysis, venous blood samples were drawn within the first 24 h of bone marrow transplant ward admission. The samples were centrifuged at 3000 rpm for 10 min at 4º C to obtain serum. Serum samples were aliquoted and quickly frozen at − 80º C until the biochemical analysis, with exception for ESR which was measured immediately.

### Statistical analysis

All statistical analyses were performed using the SPSS software (Version 20; IBM Corp., Armonk, NY, USA). Data were expressed as mean ± SD and frequency (percentage) for continuous and categorical variables, respectively. To assess differences in continuous variables between patients with and without nutritional risk, independent sample t-test was used. Also, we used the chi-square test to assess distribution of categorical variables across patients with and without nutritional risk. To compare multivariable-adjusted means of inflammatory biomarkers, albumin, and total protein between patients with and without nutritional risk, we used one-way analysis of covariance (ANCOVA). In this analysis, adjustments were made for age, gender, BMI, malignancy type, serum levels of magnesium, calcium, and total bilirubin to obtain an independent association. Both continuous and categorical confounding variables were considered as covariates in the ANCOVA analysis. P < 0.05 was considered as statistical significance.

## Results

**Fig. 1:**
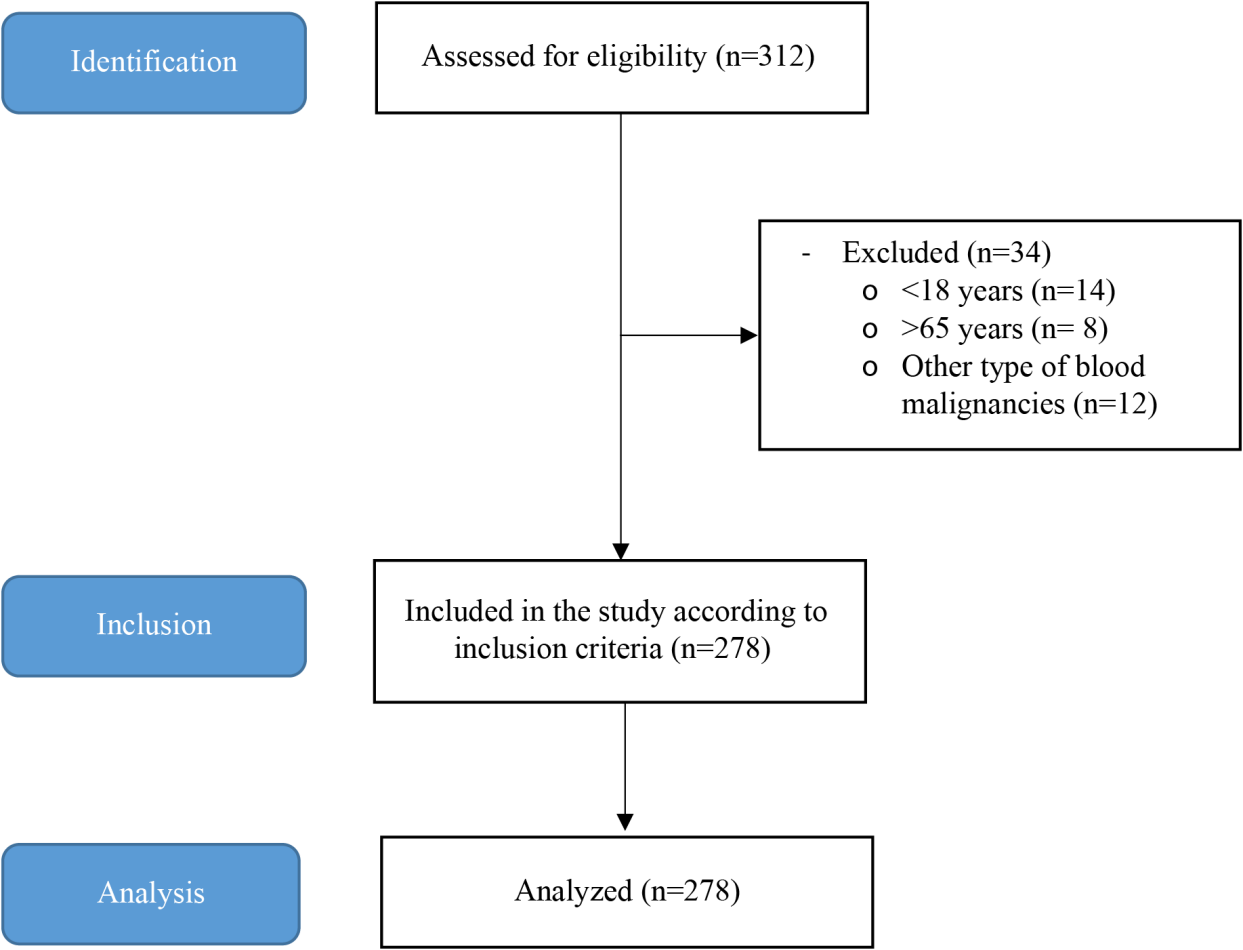
Flow chart of the study design

At first, 312 subjects were screened for the current study; however, 278 patients including 161 male (57.9%) met the inclusion criteria. Flow chart of the study design is presented in Fig. 1. The prevalence of nutritional risk (NRS ≥ 3) was 22.3%. Demographic and clinical characteristics of the patients are presented in Table 1. As shown, the mean age (± SD) of the patients was 41.46 ± 14.67 years. Also, the average of BMI was significantly lower among undernourished patients (P < 0.001). Regarding the type of malignancies, the most prevalent type was MM (35.6%); however, the prevalence of nutritional risk was higher among the patients with AML in comparison with the other types (30.6%).

**Table 1:** Demographic and clinical characteristics of patients with malignancy according to NRS scores

	Characteristics	Total (n = 278)	NRS < 3 (n = 216)	NRS ≥ 3 (n = 62)	P-value*
	Age (year)	41.46 ± 14.67	42.55 ± 14.52	37.66 ± 14.64	0.02
	Sex (male)	161 (57.9)	119 (55.1)	42 (67.7)	0.07
	Weight (kg)	76.88 ± 14.76	79.09 ± 13.87	69.17 ± 15.29	< 0.001
	BMI (kg/m^2^)	26.93 ± 4.70	27.84 ± 4.23	23.73 ± 4.89	< 0.001
**Type of malignancy**, n (%)					0.008
	MM, n (%)	99 (35.6)	85 (39.4)	14 (22.6)	
	HL, n (%)	55 (19.8)	45 (20.8)	10 (16.1)	
	NHL, n (%)	28 (10.1)	23 (10.6)	5 (8.1)	
	AML, n (%)	61 (21.9)	42 (19.4)	19 (30.6)	
	ALL, n (%)	35 (12.6)	21 (9.7)	14 (22.6)	

Data are presented as mean ± SD or number (percent)

Abbreviations: BMI: Body Mass Index; MM: Multiple Myeloma; HL: Hodgkin Lymphoma; NHL: Non-Hodgkin Lymphoma; AML: Acute Myeloid Leukemia; ALL: Acute Lymphocytic Leukemia, NRS: Nutrition Risk Screening

*Obtained from the independent sample t-test or Chi-square test, where appropriate

The laboratory parameters of the patients are summarized in Table 2. As presented, there was no significant difference between the baseline laboratory parameters between patients with and without nutritional risk except for RBC (P = 0.001), hematocrit (P = 0.02), and prothrombin time (PT) (P = 0.03). The patients at nutritional risk had lower RBC, hematocrit, and higher prothrombin time compared with those without nutritional risk.

**Table 2:** Laboratory parameters of the patients with malignancy according to NRS scores

	Characteristics	Total (n = 278)	NRS < 3 (n = 216)	NRS ≥ 3 (n = 62)	P-value*
**CBC**					
	RBC (Million/µl)	4.07 ± 0.74	4.14 ± 0.71	3.80 ± 0.77	0.001
	WBC (×10^3^/µl)	5.12 ± 1.79	5.12 ± 1.72	5.13 ± 2.02	0.97
	Platelet (×10^3^/µl)	189.21 ± 67.16	189.88 ± 65.66	186.88 ± 72.67	0.75
	Hgb (g/dL)	11.86 ± 1.92	12.00 ± 1.78	11.37 ± 2.30	0.05
	Hct (%)	34.71 ± 5.45	35.11 ± 5.24	33.34 ± 5.98	0.02
**Electrolyte status**					
	Na (mEq/L)	142.07 ± 3.16	142.21 ± 3.15	141.58 ± 3.19	0.16
	K (mEq/L)	4.13 ± 2.12	4.17 ± 2.40	4.00 ± 0.34	0.57
	Ca (mg/dL)	9.55 ± 1.00	9.54 ± 1.08	9.61 ± 0.65	0.61
	P (mg/dL)	3.94 ± 0.63	3.91 ± 0.59	4.01 ± 0.75	0.27
	Mg (mEq/L)	1.93 ± 0.23	1.92 ± 0.23	1.98 ± 0.23	0.11
**Liver function tests**					
	ALT (U/L)	32.97 ± 17.68	32.89 ± 17.67	33.27 ± 17.86	0.88
	AST (U/L)	26.79 ± 10.03	26.49 ± 9.38	27.83 ± 12.03	0.35
	ALP (U/L)	208.55 ± 67.60	205.53 ± 67.81	219.06 ± 66.35	0.16
	Bilirubin-T (mg/dL)	0.91 ± 1.73	0.95 ± 1.96	0.79 ± 0.31	0.54
	Bilirubin-D (mg/dL)	0.30 ± 0.14	0.30 ± 0.14	0.31 ± 0.14	0.67
**Renal function tests**					
	BUN (mg/dL)	14.03 ± 4.11	14.18 ± 4.00	13.50 ± 4.48	0.26
	Cr (mg/dL)	0.94 ± 0.17	0.95 ± 0.17	0.91 ± 0.14	0.13
**Coagulation tests**					
	PT	12.20 ± 0.90	12.14 ± 0.89	12.42 ± 0.91	0.03
	PTT	31.02 ± 5.58	30.71 ± 5.47	32.09 ± 5.85	0.08
	INR	1.10 ± 0.86	1.11 ± 0.98	1.03 ± 0.07	0.51

Data are presented as mean ± SD

Abbreviations: CBC: Complete Blood Count; RBC: Red Blood Cell; WBC: White Blood Cell; PLT: Platelet; Hgb: Hemoglobin; Hematocrit; Na: Sodium; K: Potassium; Ca: Calcium; P: Phosphor; Mg: Magnesium; ALT: Alanine Aminotransferase; AST: Aspartate Aminotransferase; ALP: Alkaline Phosphatase; BUN: Blood Urea Nitrogen; Cr: Creatinine; PT: Prothrombin time; PTT: Partial Thromboplastin Time; INR: International Normalized Ratio

*Obtained from the independent sample t-test

The association between nutritional status based on NRS-2002 and inflammatory biomarkers as well as albumin and total protein are shown in Table 3. Compared with patients with normal nutritional status, those with nutritional risk (NRS ≥ 3) had higher ESR, CRP, and CAR. These differences remained significant even after adjustment for potential confounders. In addition, undernourished patients had lower BMI, serum albumin, and hemoglobin concentration compared with those without nutritional risk (P < 0.05). We found no significant difference in total protein between patients with and without nutritional risk either before or after adjusting for confounders (P > 0.05).

**Table 3:** Multivariable-adjusted means for inflammatory biomarkers, albumin and total protein across the two categories of NRS

		NRS < 3 (n = 216)	NRS ≥ 3 (n = 62)	P-value^*^
BMI (kg/m^2^)				
	Crude	27.84 ± 0.29	23.73 ± 0.55	< 0.001
	Model 1	27.75 ± 0.29	24.05 ± 0.55	< 0.001
	Model 2	27.77 ± 0.29	23.98 ± 0.55	< 0.001
CRP (mg/L)				
	Crude	8.93 ± 1.14	15.37 ± 2.14	0.009
	Model 1	8.96 ± 1.15	15.27 ± 2.25	0.02
	Model 2	8.90 ± 1.15	15.47 ± 2.26	0.01
ESR (mm/hr)				
	Crude	23.10 ± 1.55	28.01 ± 2.78	0.12
	Model 1	22.60 ± 1.52	29.61 ± 2.85	0.03
	Model 2	22.37 ± 1.53	30.35 ± 2.88	0.01
Albumin (g/dL)				
	Crude	4.50 ± 0.02	4.32 ± 0.05	0.002
	Model 1	4.51 ± 0.02	4.29 ± 0.05	< 0.001
	Model 2	4.51 ± 0.02	4.28 ± 0.05	< 0.001
Total protein (g/dL)				
	Crude	7.09 ± 0.06	7.08 ± 0.11	0.94
	Model 1	7.08 ± 0.06	7.09 ± 0.11	0.97
	Model 2	7.09 ± 0.06	7.08 ± 0.11	0.98
Hemoglobin (g/dL)				
	Crude	12.00 ± 0.13	11.37 ± 0.24	0.02
	Model 1	12.00 ± 0.12	11.39 ± 0.24	0.03
	Model 2	12.02 ± 0.12	11.32 ± 0.24	0.01
CRP/Alb				
	Crude	2.02 ± 0.27	3.68 ± 0.52	0.005
	Model 1	2.03 ± 0.28	3.65 ± 0.54	0.01
	Model 2	2.02 ± 0.28	3.70 ± 0.55	0.008

Data are presented as mean ± SD

Abbreviations: CRP: C-reactive protein, ESR: Erythrocyte Sedimentation Rate, NRS: Nutrition Risk Screening

Obtained from one-way analysis of covariance

Model 1: Adjusted for age, gender, BMI, and malignancy type

Model 2: Further adjustment for serum levels of magnesium, calcium, and total bilirubin

## Discussion

In clinical practice, several biochemical parameters, particularly albumin, are often used for assessment of nutritional risk; however, there is still a lack of evidence to support their usefulness under special clinical circumstances [20]. To the best of our knowledge, this is the first study to explore the association between nutritional status and several blood biomarkers and systemic inflammation in patients with hematological malignancies. In this study, we found a significant positive association between nutritional risk and some inflammatory biomarkers including ESR, CRP, and CAR and a significant inverse association between nutritional risk and BMI, serum albumin, and hemoglobin concentration in patients with hematological malignancies after taking potential confounders into account.

According to our results, 22.3% of HSCT candidates were at nutritional risk. The extent of nutritional risk in these patients depends on several factors including pretreatment of high-dose chemotherapy and/or systemic radiation before transplantation, which may cause important metabolic alterations, digestive dysfunction, and nutritional deficiencies [21]. Although nutritional risk has been identified as a serious condition under many circumstances, there is still a lack of universal approach and method for nutritional assessment. Several nutritional assessment tools such as NRS-2002, PG-SGA and MNA have been developed as valid and successful approaches in clinical setting [18, 22]. Of these, the ESPEN recommends the use of NRS-2002, which includes the diagnosis of cancer as a risk factor for poor nutritional status, in hospitalized patients. Although according to the ESPEN guideline of 2017, there is no consensus on the nutritional screening methods for cancer patients [23–25], Peng et al., suggested NRS-2002 as the first choice of nutritional assessment tools for patients with leukemia before HSCT [26].

The results of the present study showed a positive association between nutritional risk and CAR and inflammatory biomarkers including CRP and ESR, and a significant inverse association between nutritional risk and albumin and hemoglobin. However, no significant association was seen for total protein. In agreement with our results, previous studies have shown that nutritional risk (based on NRS-2002) was positively associated with inflammation [27–29] and CAR [28], and was inversely associated with albumin [27, 28, 30–33] and hemoglobin levels [27–30, 33]. A recent cross-sectional study among hospitalized patients reported a significant association between nutritional risk (based on NRS-2002) and lower albumin [34]. Also, the findings from a systematic review and meta-analysis in older adults revealed a significant association between NRS score and albumin and hemoglobin levels in acute and non-acute patients; however, this association was not significant regarding to CRP levels [20].

With respect to the association between NRS score and blood biomarkers, the present study demonstrated that there is a strong association between nutritional status and serum albumin levels (P < 0.001), while others did not. This could be in large part due to albumin being a more sensitive marker, reflecting poor nutritional assessment and inflammation in HSCT candidates. In addition, nutritional risk has been identified as an important contributing factor to the development of inflammation; however, inflammation is expected to be common among HSCT candidates [6] and this is confirmed by increased serum level of CRP and ESR values in our study. Although inadequate nutritional intake can lead to a decrease in serum albumin, inflammation may also affect the nutritional indices. Nutritional risk is associated with compromised immunity and an increased chance of infection [35]. This condition can induce inflammation among malnourished patients [36]. However, it must be kept in mind that albumin is one of the negative acute-phase proteins (APP) that are decreased by increasing inflammatory biomarkers [37, 38]. Therefore, the increased levels of inflammatory biomarkers, rather than nutritional risk, may lead to reduced levels of albumin. Hence, our findings on the significant association between nutritional status and serum albumin levels should be considered with caution. Overall, the combination of both nutritional risk and inflammation, as occurred in hematological malignancies, results in great changes such as poor transplant outcomes [39, 40].

We also found a significant association between nutritional risk and lower BMI in line with earlier studies [27, 28, 30, 31, 33]. In contrast, Boban et al. reported no significant association between NRS score and BMI [41]. Considering that the BMI is a component of NRS tool, it was not the focus of our study as a marker; however, given the widespread utilization in clinical practice, it was worth to present its results here. Our results showed even though the patients’ BMI might be in the normal range, they may be at nutritional risk, suggesting the necessity of using a higher cut-off for BMI to warrant identification of all at-risk individuals for our study population. So, it remains a challenge to define a practical and valid cut-off for BMI [42].

The current study had several strengths and limitations. The main strength was its homogenous population of study and evaluation of the patients before undergoing chemotherapy and transplantation which may affect nutritional status and blood biomarkers. According to our knowledge, this is the first study investigating the association between nutritional status and blood biomarkers and systemic inflammation among HSCT candidates. The study’s cross-sectional design was the main limitation and hence, follow-up surveys evaluating the effect of nutritional status on transplant outcomes and mortality are needed. In addition, body weight and BMI are traditional parameters for assessment of nutritional status. Recently, body composition analysis (BIA) has been identified as a useful and valid method in clinical practice [43]; however, it was not available in the present study.

## Conclusions

In summary, we found a significant positive association between nutritional risk and some inflammatory biomarkers including ESR, CRP, and CAR and a significant inverse association between nutritional status and BMI, serum albumin, and hemoglobin concentration in patients with hematological malignancies However, in terms of serum total protein, no significant association was seen. Further research is needed to evaluate the association between nutritional status and other outcomes of hematological malignancies such as infection, graft versus host disease (GvHD), and mortality.

## Data Availability

The datasets analyzed during the current study available from the corresponding author on reasonable request.
